# Boundary spanning at the science–policy interface: the practitioners’ perspectives

**DOI:** 10.1007/s11625-018-0550-9

**Published:** 2018-03-27

**Authors:** A. T. Bednarek, C. Wyborn, C. Cvitanovic, R. Meyer, R. M. Colvin, P. F. E. Addison, S. L. Close, K. Curran, M. Farooque, E. Goldman, D. Hart, H. Mannix, B. McGreavy, A. Parris, S. Posner, C. Robinson, M. Ryan, P. Leith

**Affiliations:** 1 The Pew Charitable Trusts, Washington, DC, USA; 2 Luc Hoffmann Institute, WWF International, Gland, Switzerland; 3 College of Forestry and Conservation, University of Montana, Missoula, Montana USA; 4 Centre for Marine Socioecology, University of Tasmania, Hobart, Tasmania Australia; 5 Center for Community and Citizen Science, University of California, Davis, California USA; 6 Climate Change Institute, Australian National University, Canberra, Australia; 7 Department of Zoology, University of Oxford, Oxford, UK; 8 Consortium for Science, Policy and Outcomes, Arizona State University, Tempe, Arizona USA; 9 COMPASS Science Communication, Portland, Oregon USA; 10 Senator George J. Mitchell Center for Sustainability Solutions, School of Biology and Ecology, University of Maine, Orono, Maine USA; 11 Department of Communication and Journalism, Senator George J. Mitchell Center for Sustainability Solutions, University of Maine, Orono, Maine USA; 12 Science and Resilience Institute at Jamaica Bay, City University of New York, Brooklyn College, New York, New York USA; 13 Gund Institute for Environment, University of Vermont, Burlington, Vermont USA; 14 Engaging Scientists and Engineers in Policy, Washington, DC, USA; 15 University of Cambridge Conservation Research Institute, Cambridge, UK; 16 Tasmanian Institute of Agriculture, School of Land and Food, University of Tasmania, Hobart, Tasmania Australia

**Keywords:** Boundary organizations, Boundary spanning, Science-policy interface, Wicked problems, Sustainability

## Abstract

Cultivating a more dynamic relationship between science and policy is essential for responding to complex social challenges such as sustainability. One approach to doing so is to “span the boundaries” between science and decision making and create a more comprehensive and inclusive knowledge exchange process. The exact definition and role of boundary spanning, however, can be nebulous. Indeed, boundary spanning often gets conflated and confused with other approaches to connecting science and policy, such as science communication, applied science, and advocacy, which can hinder progress in the field of boundary spanning. To help overcome this, in this perspective, we present the outcomes from a recent workshop of boundary-spanning practitioners gathered to (1) articulate a definition of what it means to work at this interface (“boundary spanning”) and the types of activities it encompasses; (2) present a value proposition of these efforts to build better relationships between science and policy; and (3) identify opportunities to more effectively mainstream boundary-spanning activities. Drawing on our collective experiences, we suggest that boundary spanning has the potential to increase the efficiency by which useful research is produced, foster the capacity to absorb new evidence and perspectives into sustainability decision-making, enhance research relevance for societal challenges, and open new policy windows. We provide examples from our work that illustrate this potential. By offering these propositions for the value of boundary spanning, we hope to encourage a more robust discussion of how to achieve evidence-informed decision-making for sustainability.

## Introduction

Scientific research has a key role to play in developing sustainability solutions. However, integrating science into decision-making processes about sustainability (or any complex or “wicked” issue) alongside the many actors, institutions, types of knowledge, jurisdictions, political processes, and other social issues remains a significant challenge (Cook et al. 2010; McCright and Dunlap 2011; Cvitanovic et al. 2014; Addison et al. 2015; Hering 2015; Cairney 2016; Clark et al. 2016a). Yet, effective solutions have to account for this tangle of overlapping and shifting issues. Moreover, sustainability challenges cannot wait for a slow diffusion of solutions from the scientific community that may or may not be useful (Kates et al. 2001). Indeed, the United Nations Foresight report ranked “Re-connecting Science to Policy” as the fourth out of 21 top challenges for sustainability in the twenty-first century (UNEP 2012). This is one voice in a chorus of calls over the last few decades to update and re-shape what constitutes useful science for highly complex social problems such as sustainability (Funtowicz and Ravetz 1993; Lubchenco 1998; Gibbons 1999; Guston 2004; Fazey et al. 2018). This goes beyond the ability of scientists to communicate their research findings more eloquently. Instead, these calls emphasize finding ways for society to “speak back to science” (Gibbons 1999).

In response, there has been considerable academic interest in identifying principles and processes that might build a more dynamic relationship between science, policy, and society (e.g., Kates et al. 2001; Cash et al. 2003; Clark et al. 2016a; Dilling and Lemos 2011; Lemos et al. 2012; Miller et al. 2014; Cvitanovic et al. 2016; Marshall et al. 2017; Fazey et al. 2018). More recently, practitioners of many kinds have been trying to build on those principles (e.g., Guston 2001; McGreavy et al. 2013; Reed et al. 2014; Bednarek et al. 2015, 2016; Wyborn 2015; Clark et al. 2016b). However, the emergence of these “boundary spanners”—organizations and individuals that work specifically in the science–policy interface—may be outpacing our understanding of how best to enable effective relationships between science and policy in practice.

Some recent efforts have sought to address the challenge of creating feasible and effective practices for boundary spanning (e.g., Bednarek et al. 2016). To build on these, in May 2017, The Pew Charitable Trusts convened a meeting of boundary-spanning practitioners (the authors) focused on sustainability and environmental issues to (1) reflect on progress to date, (2) develop a value proposition for boundary-spanning activities, and (3) identify the challenges and opportunities to mainstreaming boundary-spanning activities more broadly. The participants represented more than 130 years of cumulative practical experience operating at the interface of science and decision-making in 11 countries. This perspective represents the outcomes of this workshop. In sharing our experiences, we hope to contribute a practitioners’ perspective to the discourse regarding the relationship between science, policy, and society.

We present our perspective in three parts. First, we articulate our shared definition of the practice of boundary spanning and the types of activities it encompasses. As part of this discussion, we identify the core features of boundary spanning that distinguish it from other approaches to improving the use of research in policy, including communicating more effectively about research results (science communication), addressing socially-relevant research questions based on a researcher’s conception of usefulness (applied science), or advocating for policy changes that reflect research results (advocacy). Second, we propose specific value propositions for boundary spanning based on our observations practicing it. Finally, we reflect on opportunities to more effectively bring these activities to the fore of mainstream research, training, and funding efforts.

Although the ideas presented in this paper are focused on the role of scientific research in policy (because that is where the workshop participants have the most experience), we recognize the importance of considering science in conjunction with other kinds of knowledge relevant to a decision-making process (e.g., traditional knowledge). Indeed, as boundary spanners, we often account for multiple interests and sources of knowledge, recognizing that decision makers rarely use research evidence in isolation. In addition, although the workshop participants have worked throughout the world, the examples we discuss in this perspective are mainly from western contexts. We recognize, however, that approaches to integrate science and policy are highly context specific (across both space and time) and have unique opportunities and challenges in different geographic settings. We encourage other practitioners, working with different kinds of knowledge in different cultural settings and sectors, to build on our efforts and share their experiences.

## What is boundary spanning?

Boundary spanning as a concept first emerged in the 1970s in the business and organizational management literature which sought to identify organizational characteristics (e.g., specific functions or roles) that facilitate knowledge exchange between two or more organizations (e.g., Aldrich and Herker 1977; Leifer and Delbecq 1978). More recently, the importance of constructive knowledge exchange has been taken up by those trying to understand how to address “wicked problems” or complex social challenges such as sustainability (Guston 2001; Brown et al. 2010). The idea is that solutions for wicked problems have to account for many dimensions of “knowing and learning” (Kates et al. 2001). This includes the ways different actors engaged in, or affected by, an issue view the cause of a problem, their institutional and political incentives, how they feel about each other, how they view the relevance and credibility of available evidence, how they access and understand evidence, and how they view potential solutions and their viability. Indeed, solutions generated without accounting for all these moving parts are not likely to align with the information needs within a decision process.

Drawing on these features, the academic literature, and our collective experiences, we define the practice of boundary spanning as ‘work to enable exchange between the production and use of knowledge to support evidence-informed decision-making in a specific context’ and boundary spanners ‘as individuals or organizations that specifically and actively facilitate this process’. Essentially, boundary spanners dedicate their time to creating and enabling effective knowledge exchange. We recognize that knowledge production and use are not immutable categories; individuals and organizations can play multiple or shifting roles in producing or using knowledge within the same process (e.g., a decision maker who uses research in their decision-making could also provide knowledge about an issue). We also note that boundary spanning is more than just a one-to-one matching process between production and use (e.g., it involves more than just a decision maker articulating a specific question or need). As we have described earlier, accounting for the broader context of actors, perspectives, values, contested evidence, decision-making history, and power dynamics is critical in shaping a productive knowledge exchange process. We contend that with sufficient time, resources, and expertise dedicated to it, boundary spanning can sustain productive interactions between science, policy, and society, lead to increasingly useful science, and ultimately build capacity for science to inform decision-making about sustainability. While some may prefer terms other than “boundary spanning,” we use it as a starting point for a discussion of the practice of connecting science and policy.

Our definition of boundary spanning encompasses a spectrum of roles and organizational configurations. In some cases, an individual researcher can act as a boundary spanner and work to understand and reflect user needs in their research program, as well as to create opportunities for themselves to engage in a decision-making process. Given the intensity and scope of the work required, however, we have found that boundary spanners are more likely to act in a full-time capacity as an expert intermediary, rather than being engaged directly in research, or to work within a team of researchers and boundary spanners to create integrated, solution-based research programs. In Table 1, we provide examples of a variety of boundary spanners and organizations, each with different boundary functions. For example, an individual could work with a research institute to help researchers to reflect user needs in their research programs and facilitate effective policy engagement (Cvitanovic et al. 2017). At the level of organizations, teams of boundary spanners may work together as a collective to divide the work into more manageable parts and fulfill different needs within the knowledge exchange process (e.g., California Ocean Science Trust). In other cases, a team of boundary spanners may focus on building capacity among scientists and decision makers to engage in boundary spanning (e.g., COMPASS; Smith et al. 2013). In yet other configurations, university-based centers focus on solution-driven collaborations of teams of researchers and boundary spanners who can engage with users and develop relevant research (e.g., Mitchell Center; Hart et al. 2015). Some funding agencies, through their grant-making, actively match the production of science with specific decision-making needs and context using boundary spanners (e.g., the Lenfest Ocean Program; Bednarek et al. 2015).

**Table 1:** Examples of boundary spanners and organizations, and their boundary-spanning functions

Boundary individual, program, or organization	Examples of boundary-spanning functions	References
CSIRO Climate Adaptation Flagship knowledge broker, Australia	Knowledge broker coordinates policy scanning and engagement for researchers, trains researchers in stakeholder engagement and outreach, helps researchers understand policy processes and decision-making institutions	Cvitanovic et al. (2017)
AAAS Science and Technology Fellows, USA	The program places PhD level scientists in policy settings, primarily within the U.S. Scientists in this policy fellowship may be acting as decision makers or supporting them, but either way, may scope political and policy processes and how different actors view and understand available research	https://www.aaas.org/page/fellowships; AAAS (2017)
COMPASS, USA	COMPASS acts as a boundary-spanning practitioner to facilitate more scientists engaging effectively in the public discourse about the environment. Through communication trainings, coaching and real-world connections, they support researchers to build the communication skills, networks, and relationships they need to realize this vision. They are a non-profit, non-advocacy organization	https://www.compassscicomm.org/
Luc Hoffmann Institute, Switzerland	The institute was set up as an independent research center with mandate to provide fresh perspectives on critical conservation challenges. The Institute aims to convene dialogues, facilitate new ways of thinking that build on diverse perspectives and translate ideas into action	http://luchoffmanninstitute.org
California Ocean Science Trust, USA	Independent non-profit created through state legislation to improve collaborations between scientists and decision makers. The staff develop synthesis products, facilitate collaborative processes, align and secure funds on priority areas, and collaboratively develop strategies for connecting science and policy	Pietri et al. (2011); Meyer et al. (2015); CORSA (2000)
Mitchell Center, University of Maine, USA	The center supports interdisciplinary research teams that work in long-term, iterative collaborations with decision makers and other stakeholders. Teams include experts in engagement and co-production. Institutional capacities include policy scanning, serving as an honest broker, convening stakeholders, facilitating researcher–practitioner interactions, managing internal and external conflicts, and obtaining research funding	Hart et al. (2015), McGreavy et al. (2013)
The Lenfest Ocean Program, Washington, DC, USA	The Program supports policy-relevant research grants. Staff scan relevant policy and science contexts to assess policy-relevant research questions about ocean ecosystems. Staff facilitate engagement and communication between researchers, decision makers, and other relevant parties (through active partnerships or regular consultations) to develop and support research that can address policy needs, and ensure, throughout the research process, that the research continues to address decision-maker needs and informs decision-making processes	http://lenfestocean.org; Bednarek et al. (2015)
Regional Integrated Science and Applications Program (RISA), USA	Federal funding from the National Oceanic and Atmospheric Administration (NOAA) supports regionally focused research centers that coproduce relevant and useful climate science products, working directly with stakeholder groups in an end-to-end process that meets their needs, and helps to build resilience and adaptive capacity	Parris et al. (2016)

While components of boundary spanning are similar to other roles at the interface between science and decision-making (e.g., science communication, applied science, and advocacy), we believe that several features help distinguish it as a distinct practice. First, boundary spanners recognize that effective communication is necessary but not sufficient in connecting science and policy. Instead, they tend to focus on interactive and regular exchanges aimed at understanding what research would be most useful and why, and how other actors and sources of knowledge factor into the decision-making process, rather than packaging research for transmission to potential “users” (Cvitanovic et al. 2015a). These iterative exchanges help refine the mutual understanding about research questions and the type of research that is most needed. They also help build the relationships and broader social formations that are necessary to facilitate the uptake of that research (Jasanoff 2004). These exchanges also differentiate boundary spanning from applied science. While research aimed at solving specific problems is a critical part of generating sustainability solutions, applied researchers do not typically have the resources (e.g., time, money, etc.) to actively engage users in developing or implementing research in an iterative way (e.g., Cvitanovic et al. 2016). Third, rather than acting as advocates for specific research results or policy changes, boundary spanners aim to foster trust that they, and in many cases, the scientists and others with whom they work are not pushing an agenda or choosing research findings to fit a particular position (Lacey et al. 2018). In this way, they strive to act in accordance with Pielke Jr’s (2007) description of an honest broker, whereby they do not advocate for a single cause or predetermined outcome. Instead, they aim to consider and offer multiple available options and perspectives, and to cultivate a process of knowledge creation and exchange that can be viewed as rigorous, credible, and legitimate (Cash et al. 2003). We are not implying that boundary spanners are value-free and neutral. However, they aim to be reflective and comprehensive about identifying perspectives and values within a process, including their own and those of the scientists involved, so that those values are explicitly recognized and accounted for whenever possible.

## A value proposition for boundary spanning

We view boundary spanning as a distinct and emerging practice. Thus, we believe it is useful to understand the mechanisms by which it contributes to more productive relationships between science and policy, both to improve its practice and understanding of its role in knowledge exchange. In this section, we outline four potential benefits of dedicating time and expertise to boundary spanning and illustrate these by drawing on examples from our collective body of work. We developed these from reflections during the workshop on our experiences as boundary spanners.

First, our experiences suggest that regular and sustained boundary spanning can help increase the efficiency by which scientific research is tailored for consideration within decision-making. Our observations within the sustainability sector, as well as within other sectors (e.g., education), suggest that research “designed for action” targeted for specific contexts is more likely to be considered in decision-making (see Mosley and Courtney 2012; Bogenschneider et al. 2010; Goertz et al. 2013). By creating a system for effective knowledge exchange and dedicating time to scanning relevant scientific research and policy issues, boundary spanners can help track current and emerging science needs in decision-making to help the scientific community focus research efforts accordingly (McNie 2007; Sarewitz and Pielke 2007). For example, in research projects associated with the Mitchell Center for Sustainability Solutions in Maine, US, team members often found that it was essential to work with intended decision makers throughout a research process to ensure that the research questions were relevant and those who might use the results were involved in ways that would support their ability to eventually to do so. This group found that having a system for assessing research needs and preferences for partnerships supported effective tailoring of research question and design (Bieluch et al. 2017). We propose that these efforts can also reduce the risks of science not meeting decision maker needs and mismatches between the timing of research and decision making, or decisions moving forward without sufficient evidence to inform them. An example of this can be seen in the Australian context of the Cooperative Research Centres (CRCs), specifically the eWater CRC. Through consistent boundary spanning, such as brokering between science and policy and communication management, the project was able to adapt research focus areas and align decision support tool development with both national and state level policy needs to support the development of formal policy instruments for the health of Great Barrier Reef (Carroll et al. 2012).

Second, we suggest that boundary spanning can increase the potential for durable decision processes and policy. This is not to say that the goal is to support decisions that are static or unchangeable. Rather, we mean decision-making processes that can integrate new evidence and perspectives, including through periods of change, such as changes in governance or unexpected conflicts that may arise (recognizing that political processes can change decision-making dynamics at any point). We contend that boundary spanners can support process durability in two ways. First, boundary spanners’ focus on facilitating knowledge exchange means that they assess how different actors understand and process information, and aim to cultivate meaningful, trusted and sometimes sustained relationships among those involved. Based on our experience, we suggest that creating and nurturing this knowledge exchange infrastructure can help actors in the process (including scientists) absorb new information and account for conflicting evidence without derailing an entire process. Similarly, boundary spanners aim to identify and account for contradictory evidence and divergent perspectives as early in the process as possible. We suggest that this comprehensive scanning function may help manage the risk that either a single ideology will shape policy, leaving it likely to be dismantled to meet a different agenda, or that contradictory scientific or other knowledge will reverse a decision.

Third, we suggest that dedicated boundary spanning can help increase the legitimacy (Cash et al. 2003) and social robustness (Gibbons 1999) of science, or the degree to which science is accepted among a diverse set of actors and is relevant for societal challenges. Boundary spanners specifically aim to increase permeability between science and policy to promote “testing and retesting” of the usefulness of scientific knowledge (see Gibbons 1999). Our experiences suggest that boundary spanning can result in those involved better understanding the role and value of multiple sources of knowledge, including science, and feeling that their perspectives have been considered. We contend that this could decrease the potential for science to be seen as a vehicle for pushing a particular viewpoint at the expense of other perspectives (and in turn, decreasing the potential for contesting it). We propose that this focus on legitimacy may also help to mitigate the politicization of science, which can be aggravated by scientists advocating for their “rightful” role or favorite research findings (Sarewitz 2016). COMPASS, a boundary organization in the U.S., for example, focuses on empowering scientists to directly share what they know, without making direct policy recommendations. COMPASS boundary spanners coach scientists to be ready for the “what should we do?” question that inevitably comes from policymakers, by arming them to explain how science can help make the implications of a range of policy options more transparent. Scientists see close up how their science can be used in decisions and they become more open to sharing the substance of what they know without an agenda. As a result, scientists become trusted resources and connectors to others with relevant knowledge in their academic communities. This is particularly pertinent at the moment given a growing tension between the public questioning of the value of science in public discourse and the urgency within the scientific community to ensure its status and role in decision-making (e.g., McCright and Dunlap 2011; Gillard 2016).

Fourth, we contend that, by comprehensively scanning the policy context, boundary spanners can identify current and emerging opportunities for science to inform policy, i.e., policy windows. This, in turn, may increase the opportunities for decision makers, scientists, and others to identify if, when, and how research may be able to meet a decision-making need or fit within a local context (e.g., Gibbons 1999; Cvitanovic et al. 2017; Kettle et al. 2017). Indeed, as described by Rose et al. (2017), the ability to create and capitalize quickly on new policy windows significantly increases the likelihood that a decision will be evidence informed. Finally, if the relationship-building function of boundary spanning is successful, new policy windows may open. This may be especially true if the relationships can be sustained (Honig et al. 2014). In our work, we have found sustained engagement processes leading to new policy windows and research and policy agenda alignment, even after “final” decisions are made. For example, the Lenfest Ocean Program has found that in some cases, grantees and resource managers are interested in continuing to collaborate even after a project has run its course. Most commonly, they express interest in identifying next steps for the research to be used within decision-making as well as follow-up research that could help decision makers.

## Mainstreaming boundary spanning

Our experiences suggest that boundary spanning can contribute to sustainability solutions. However, our experiences have also revealed that there are multiple challenges to realizing this potential, which we canvass here. This is not meant to be a comprehensive review or analysis, but rather, a list of some key challenges and opportunities workshop participants identified as pressing. Moreover, we recognize that some of these challenges and potential opportunities could vary depending on cultural context (see Kates et al. 2001). We aim for this discussion to complement other emerging efforts to raise critical issues for boundary spanning and to spur meaningful conversations about what it might take to build capacity at the science–policy interface across the globe (see Schwartz et al. 2018; Fazey et al. 2018).

First, recognize boundary spanning as a distinct practice and reconfigure organizational structures accordingly. This recommendation is based on our observation that boundary spanning is confused with, and thus implemented as part of, complementary activities such as science communication, assumed to be an activity that a scientist takes on in addition to their full-time research program, or believed to be an activity that an individual can manage for an entire organization. In our experience, this tends to hinder the essential integrative function and potential of boundary spanning by not allocating sufficient time, resources, or expertise to the effort. It can also constrain professional development and job opportunities, for example, by limiting the skills profiles sought through hiring processes. This is not to say that scientists cannot be boundary spanners in addition to being researchers. However, based on our experiences, we believe that we need a much clearer conception of who is best suited to which roles, and the time and expertise necessary for it. Not all sustainability scientists desire to fill this function, excel at it, or have the time for it. We recognize that re-configuring jobs and organizations to allow for distinct boundary roles is a significant undertaking (see Keeler et al. 2017). However, we also have a critical opportunity to re-shape our institutions to more effectively address sustainability challenges. Indeed, some research institutions are already transforming themselves by organizing around solving specific problems rather than disciplinary lines (e.g., Hart et al. 2015). We urge that the role of expert boundary spanners be a critical part of the conversations about how institutions might more effectively address sustainability challenges.

Second, develop new approaches to training and professional development that emphasize the skills needed to work at the science and policy interface. It is commonly assumed that boundary spanners will primarily emerge from the scientific community, and in many cases, that these boundary spanners will be researchers who engage at the science–policy interface in addition to their existing research efforts. As we describe above, however, working at the science–policy interface can be a full time and long-term enterprise and often requires a different skill set. This includes “practical knowledge”, or a keen ability to read social cues, facilitate diverse viewpoints, and navigate complex politics (Cairney 2016), and systems or meta-thinking rather than a singular focus on an issue (see Addison et al. 2013; Bernstein et al. 2017, Schwartz et al. 2017). However, training programs for scientists (of all career stages) to engage in the science–policy interface still tend to focus on first generating research considered high quality within academia and then improving scientists’ ability to communicate that research, and training programs aimed specifically at boundary-spanners are still emerging. Thus, at the least, training programs for scientists interested in policy engagement need to be reconfigured to reflect the realities of working at the science–policy interface. This could include, for example, understanding how to meaningfully reflect user needs in a research program. Training should also focus on scientists’ ability to think as critically about the science–policy interface as they do about their own research programs. This includes reflecting on how their values and perspectives might influence their views on the kind of research that might be useful within a policy process (Bernstein et al. 2017). Given that the time commitments and skill sets needed, however, we also suggest broadening our conception of who might act as a boundary spanners and provide appropriate training (e.g., systems thinking) for expert, and potentially full time, boundary spanners.

Third, develop and implement measures of success that appropriately account for boundary-spanning activities. At present, career progression among many boundary spanners remains tied to traditional metrics (e.g., numbers of peer-reviewed publications and citations) or expectations of significant policy change. These overlook the work undertaken by boundary spanners conflate it with advocacy, and have been shown to undermine the extent to which boundary spanning can occur (Shanley and López 2009; Cvitanovic et al. 2015b). We need to describe rigorous boundary spanning and its outcomes in more detail, even it if is challenging, for example, to assess how boundary spanning changes relationships between science and policy and helps facilitate science-informed policy deliberations (see Nutley et al. 2007).

## Conclusions

Scientific knowledge, alongside other forms of knowledge, has an important contribution to make in addressing contemporary sustainability challenges. Based on our collective experiences, we contend that boundary spanning as a distinct practice can play a critical role in facilitating this contribution, by reconciling the production and use of scientific knowledge to support sustainability policy and solutions. We believe that boundary spanning has the potential to increase the efficiency by which scientific evidence informs policy, foster the capacity to absorb new evidence and perspectives, enhance research relevance for societal challenges, and open new policy windows. By offering these propositions for the value of boundary spanning, we hope to encourage a more robust and critical conversation about how best to achieve evidence-informed decision making in practice. We encourage colleagues to test and refine our value proposition, as well as to offer new and different ones.

We have also identified a number of changes which would enhance our ability to realize the potential of boundary spanning. We do not mean to imply that everyone interested in connecting science and policy more effectively needs to be a boundary spanner. There is a wide spectrum of roles across the science–policy interface. Rather, we feel that we need to better address what functions are necessary at the science–policy interface, how these roles can best be filled, and how to provide support for them. We recognize that institutional norms and professional development conventions are difficult to shift, but without these changes, we believe that opportunities to support evidence-informed decision making and sustainability solutions will be constrained.
